# Differential expression of small RNAs under chemical stress and fed-batch fermentation in *E. coli*

**DOI:** 10.1186/s12864-015-2231-8

**Published:** 2015-12-10

**Authors:** Martin Holm Rau, Klara Bojanovič, Alex Toftgaard Nielsen, Katherine S. Long

**Affiliations:** Novo Nordisk Foundation Center for Biosustainability, Technical University of Denmark, Kogle Allé 6, 2970 Hørsholm, Denmark

**Keywords:** sRNA, RNA-seq, Fermentation, Chemical stress, MicF, RybB, OmrB, CyaR, RyhB

## Abstract

**Background:**

Bacterial small RNAs (sRNAs) are recognized as posttranscriptional regulators involved in the control of bacterial lifestyle and adaptation to stressful conditions. Although chemical stress due to the toxicity of precursor and product compounds is frequently encountered in microbial bioprocessing applications, the involvement of sRNAs in this process is not well understood. We have used RNA sequencing to map sRNA expression in *E. coli* under chemical stress and high cell density fermentation conditions with the aim of identifying sRNAs involved in the transcriptional response and those with potential roles in stress tolerance.

**Results:**

RNA sequencing libraries were prepared from RNA isolated from *E. coli* K-12 MG1655 cells grown under high cell density fermentation conditions or subjected to chemical stress with twelve compounds including four organic solvent-like compounds, four organic acids, two amino acids, geraniol and decanoic acid. We have discovered 253 novel intergenic transcripts with this approach, adding to the roughly 200 intergenic sRNAs previously reported in *E. coli*. There are eighty-four differentially expressed sRNAs during fermentation, of which the majority are novel, supporting possible regulatory roles for these transcripts in adaptation during different fermentation stages. There are a total of 139 differentially expressed sRNAs under chemical stress conditions, where twenty-nine exhibit significant expression changes in multiple tested conditions, suggesting that they may be involved in a more general chemical stress response. Among those with known functions are sRNAs involved in regulation of outer membrane proteins, iron availability, maintaining envelope homeostasis, as well as sRNAs incorporated into complex networks controlling motility and biofilm formation.

**Conclusions:**

This study has used deep sequencing to reveal a wealth of hitherto undescribed sRNAs in *E. coli* and provides an atlas of sRNA expression during seventeen different growth and stress conditions. Although the number of novel sRNAs with regulatory functions is unknown, several exhibit specific expression patterns during high cell density fermentation and are differentially expressed in the presence of multiple chemicals, suggesting they may play regulatory roles during these stress conditions. These novel sRNAs, together with specific known sRNAs, are candidates for improving stress tolerance and our understanding of the *E. coli* regulatory network during fed-batch fermentation.

**Electronic supplementary material:**

The online version of this article (doi:10.1186/s12864-015-2231-8) contains supplementary material, which is available to authorized users.

## Background

Bacteria encode hundreds of small regulatory RNA molecules with typical sizes from 50 to 300 nucleotides that regulate gene expression [1, 2]. Although some sRNAs function by binding to protein targets and sequestering their activities, the majority act via base pairing interactions with target mRNA molecules, thereby affecting their translation and/or stability. The base pairing sRNAs can be divided into *cis*- and *trans*-encoded sRNAs that are encoded just opposite or at a different chromosomal location relative to their targets, respectively. The latter group of intergenic sRNAs is characterized by short and imperfect target base-pairing interactions, multiple mRNA targets, and in some bacteria, the requirement of the RNA chaperone Hfq.

The bacterium *Escherichia coli* has been the model system for the study of sRNAs and it is therefore the organism with the most comprehensive information on sRNA function available [3]. Early approaches for sRNA identification were largely based on high abundance, sequence conservation, and protein co-purification, in particular with Hfq [4]. Systematic screens for sRNAs have focused mostly on intergenic regions and utilized computational methods [5–10], shotgun cloning strategies [11, 12] and high-density oligonucleotide probe arrays [13–15]. In recent years the application of RNA-sequencing (RNA-seq) has led to the identification of hundreds of novel transcripts in diverse bacteria. In *E. coli*, two studies have used RNA-seq approaches to identify novel sRNAs and detect sRNAs predicted previously with computational methods [16, 17].

Bacterial sRNAs are often expressed in response to changing environmental conditions and function to modulate gene expression. There are a plethora of documented connections between sRNAs and stress, where sRNAs regulate important processes in response to metabolite/nutrient, envelope/outer membrane, oxidative, iron deficiency, anaerobic and pH stress [18, 19]. Chemical stress is routinely encountered during microbial bioprocessing applications due to product toxicity [20–24] because high titers above 100 g per liter are usually required for economically viable production. Despite this, the cellular response and involvement of sRNAs in this process are poorly understood. Biobased production of chemicals is usually performed by high cell density fed-batch fermentation and complete knowledge of regulation of cellular metabolism is critical for achieving improved production. This includes sRNA-derived regulation and its effect on metabolism during the different fermentation phases, such as the fed-batch phase and the transition between exponential and stationary phases. We have used an RNA-seq approach to map the *E. coli* sRNome during chemical stress and high cell density fermentation with the aim of gaining insights into the chemical stress response and identifying sRNAs with roles in stress tolerance that have potential applications in the design and optimization of future production strains. As significant differences in growth physiology are observed between batch and fed-batch fermentation conditions, we have also studied the expression of sRNAs during these two conditions. Moreover, we have investigated the expression of 462 small RNAs, comprised of previously annotated and 253 novel transcripts, and show that a significant fraction of them are differentially expressed under chemical stress and during high cell-density fermentations.

## Results

### Experimental approach

An initial list of future building block and precursor biochemicals was compiled with inspiration from a study that aimed to identify the top value added chemicals that can be produced from biomass [25]. This analysis considered the necessary transformations to convert sugars into the building block chemicals and the further conversion of these into secondary chemicals and derivatives, as well as economic parameters including the known and potential market data for the compounds. Several other targets of commercial interest as well as some inhibitors commonly found in biomass hydrolysate were also included. A series of growth inhibition tests were performed on the *E. coli* K-12 MG1655 strain with a range of compound concentrations. The initial list was reduced, where compounds with low inhibition of bacterial growth within solubility limits, similar chemical structures and low commercial potential were excluded.

The final list of twelve compounds (Table 1) includes organic acids (acetate, succinic acid, itaconic acid, and levulinic acid), amino acids (serine and threonine), organic solvent-like compounds (butanol, 3-hydroxybutyrolactone, 1,4-butanediol, and furfural), the isoprenoid precursor geraniol and the fatty acid decanoic acid. In order to investigate the response to growth inhibiting concentrations of the chemicals and to detect compound-specific responses in *E. coli* K-12 MG1655, we chose to use the concentration of compounds that reduced the exponential-phase growth rate by 33 %. Growth inhibition experiments were performed to determine this concentration for each compound (Table 1). A wide concentration range of growth inhibition was observed, including compounds with low inhibitory effects such as succinic acid and 1,4-butanediol (inhibiting concentrations > 200 mM) and high effects such as geraniol and decanoic acid (inhibiting concentrations in the low millimolar range).

**Table 1:** The chemical compounds and growth-inhibiting concentrations used in this study

Compound	Concentration	
	g/L or % (v/v)	mM
Acetate (sodium)	7.5 g/L	55
1,4-Butanediol	4 %	452
Butanol	0.75 %	82
γ-Hydroxy-butyrolactone	1.10 %	144
Decanoic acid	0.13 %	7.3
Furfural	0.10 %	12
Geraniol	0.03 %	1.7
Itaconic acid	29 g/L	223
Levulinic acid	0.70 %	69
Serine	3 g/L	29
Succinic acid	32 g/L	271
Threonine	7 g/L	59

**Fig. 1:**
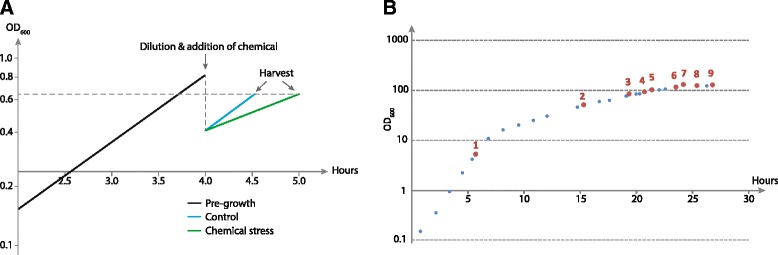
Representative growth curves of *E. coli* K12 MG1655 for the investigated conditions. **a** Chemical stress experiments were performed by addition of compound in exponential phase, followed by cell harvest after approximately one hour of growth. The stress samples were compared to a control where no compound was added and harvest performed at same OD_600_. **b** During high cell density fed batch fermentation cells were harvested at different time points along the growth curve. Measurement time points are indicated (blue circles), as well as sample time points (red circles) in exponential batch (point 1), glucose-limited exponential fed-batch (points 2–5), transition (points 6–7) and stationary (points 8–9) phases

In order to detect sRNAs, cDNA sequencing libraries were generated using RNA isolated from *E. coli* cells subjected to chemical stress or grown under high cell density fermentation conditions. For the chemical stress experiments, cells were initially grown to exponential phase, followed by compound addition, one hour of growth, and cell harvest (Fig. 1a). High cell density fermentations were performed with *E. coli* K-12 MG1655 including glucose batch and fed-batch phases, where OD_600_ values greater than 100 were reached (Fig. 1b). Cells were harvested at several points along the growth curve including exponential batch (point 1), glucose-limited exponential fed-batch (points 2–5), transition (points 6–7) and stationary (points 8–9) phases (Fig. 1b). All steps including cell growth, RNA isolation and library preparation for deep sequencing were performed in triplicate for the chemical stress samples (except those with threonine, itaconic acid and decanoic acid that were performed once) and in duplicate for the fermentation samples.

### Identification of sRNAs

A total of 217 million sequencing reads were obtained for all samples, with an average of 4.3 million reads per sample (Additional file 1). The average percentage of reads mapping to intergenic sRNAs was 18.7 % per sample, corresponding to 800,000 reads (40 million in total), while the majority of reads mapped to rRNAs. For sRNA identification, trimmed sequence reads for each sample were mapped to the *E. coli* K-12 MG1655 genome and the resulting mapping files for all samples were merged into one file containing information on all sequence reads for each library. This procedure was performed for both the fermentation and stress libraries. Expression values containing the number of reads per base pair were extracted from the mapping files, and transcript regions were identified using a cutoff of 100 reads. The transcript file was reduced to contain only transcripts residing entirely within intergenic regions using a previously designed script [26]. The expression profiles of adjacent regions abutting the intergenic transcripts were further manually curated to evaluate whether each transcript corresponded to an sRNA or untranslated region (UTR). In cases where similar expression levels and/or the lack of a gap between the transcript and adjacent gene were observed, the transcript was classified as an UTR. A total of 253 novel intergenic sRNAs were identified by this approach, adding to the 92 previously annotated sRNAs in RegulonDB 8.6 [27], and the 117 sRNAs previously detected by RNA-seq [17]. Of these groups of previously reported sRNAs, 86 % and 79 %, respectively, could be detected here. Although there was evidence of transcription for most of the other annotated sRNAs, the level was low and these were therefore classified as undetected. The novel sRNAs are denoted ES001 to ES253 based on their genomic coordinates, whereas the previously detected sRNAs are referred to either by their annotated names or the ECS designations used in an earlier RNA-seq study [17].

**Fig. 2:**
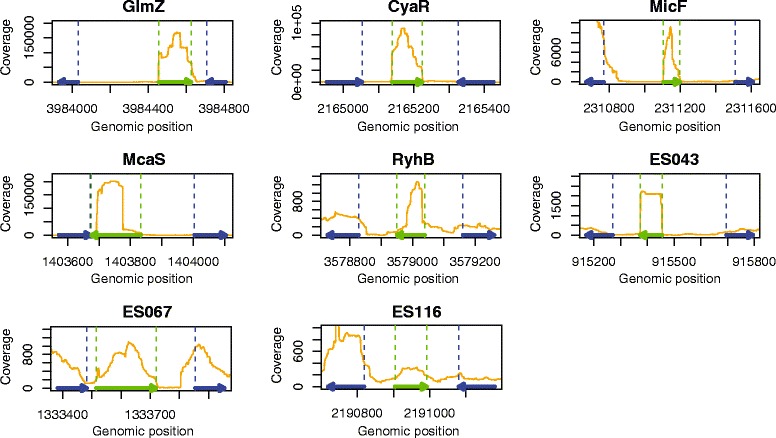
Small RNA expression profiles detected with RNA-seq. The panels include the known sRNAs GlmZ, CyaR, MicF, McaS and RyhB, a novel sRNA with clearly defined coordinates, ES043, a novel sRNA with less defined coordinates, ES067, and a novel sRNA with vaguely defined coordinates and low expression level, ES116. The y-axes of the plots denote read coverage at each nucleotide position for the pooled fermentation and stress samples. The x-axes denote the genomic positions according to the *E. coli* K-12 MG1655 genome coordinates (NC_000913.2). Legend: orange lines, expression signal; dashed green lines, sRNA coordinates; dashed blue lines, intergenic region coordinates; arrows denote directions of flanking genes (blue) and sRNAs (green)

Some representative examples of expression profiles for annotated known and novel small RNA transcripts are shown in Fig. 2. The first five plots display expression profiles in the regions containing the annotated and experimentally verified sRNAs GlmZ, CyaR, MicF, McaS, and RyhB. They demonstrate a very good correlation between actual coordinates and transcript boundaries as determined from read coverage. Although the expression profile of McaS seems to diverge from the annotated coordinates, it is actually consistent with a shorter transcript previously described in the literature [28]. The other plots illustrate expression profiles of novel sRNAs, where their coordinates can be estimated from the expression boundaries. For some sRNAs such as ES043 the boundaries are very distinct, facilitating easy estimation of sRNA coordinates, while others have less pronounced boundaries such as ES067 and ES116. The coordinates were defined based on the cutoff of 100 reads for the majority of sRNAs, whereas a subset of sRNAs chosen for further study was manually curated by visual inspection. As the actual coordinates for a few small RNAs including ES116 are not necessarily those determined through the implementation of the cutoff value, the stated coordinates for these transcripts may differ to a minor degree. The RNA-seq derived coordinates and direction for all novel and previously detected sRNAs are presented in Additional files 2 and 3, respectively.

**Fig. 3:**
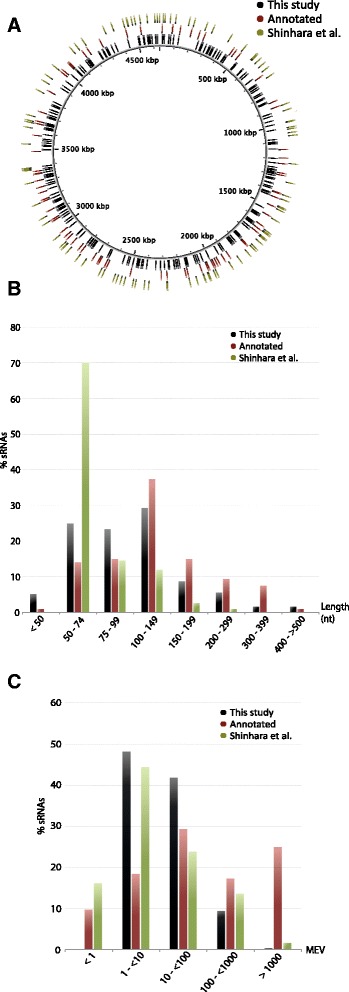
Features of the 253 novel small RNA transcripts detected in this study in relation to previously described sRNAs. Distribution of small RNAs on the *E. coli* MG1655 genome (**a**), where the novel sRNAs detected in this study are depicted on the inner ring, the known annotated sRNAs on the middle ring and the sRNAs detected in [17] on the outer ring. The transcript lengths and expression levels for the novel small RNAs compared to the known annotated and those predicted by Shinhara *et al.* [17], are shown in (**b**) and (**c**), respectively. Expression levels are presented as cumulative Mean Expression Values (MEV) defined as total stress and fermentation library reads for each sRNA divided by sRNA length

The chromosomal positions of the novel sRNA transcripts are depicted in Fig. 3a. Together with the positions of previously detected transcripts, the localization of sRNAs is evenly distributed on the genome. The lengths of the novel sRNAs were compared to previously identified transcripts as shown in Fig. 3b. Over 75 % of the novel transcripts have lengths of 50–150 nucleotides and over 95 % are shorter than 300 nucleotides. The lengths of 90 % of the annotated known sRNAs are between 50 and 300 nucleotides, where over one-third of these are in the range of 100–150 nucleotides. The other previously described sRNAs [17] are generally shorter, with over two-thirds between 50 and 75 nucleotides long, possibly due to selection of transcripts below 200 nucleotides in that study. The length distribution of annotated sRNAs is slightly skewed towards longer transcripts compared to both RNA-seq studies. This could be due to a bias in detecting longer sRNAs experimentally or that some transcripts predicted by RNA-seq are actually longer *in vivo*. The expression levels of all known transcripts, including novel transcripts and previously reported sRNAs, are represented as cumulative mean expression values (MEVs) in Fig. 3c (MEVs of each sRNA are provided in Additional files 2 and 3). Approximately 42 % of the novel transcripts have MEVs between 10 and 100 and 10 % have MEVs > 100. For the known annotated and other previously described sRNAs [17], roughly 70 % and 40 %, respectively, have MEVs of >10. The majority of highly expressed sRNAs belong to the group of annotated sRNAs, which is sensible as highly expressed sRNAs are more likely to be discovered experimentally. However a significant number also have quite low expression. This could be constitutive or limited to the particular experimental conditions investigated here.

### Differential expression of sRNAs during high cell density fermentation

**Fig. 4:**
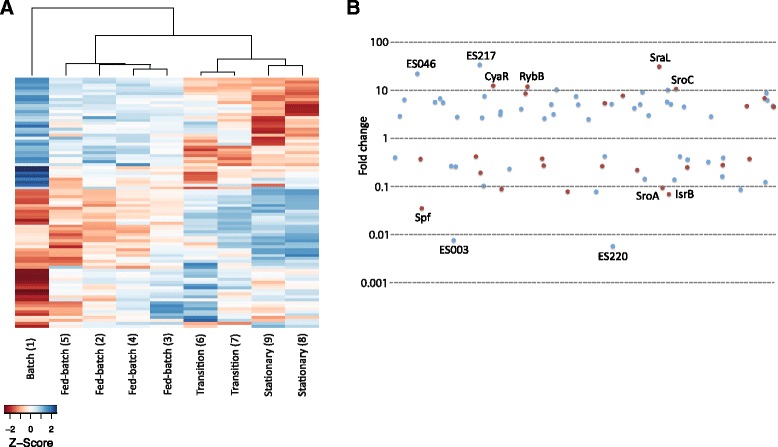
Differential expression of small RNAs during high cell density fermentation. **a** Heat map showing relative expression levels of small RNAs during high cell density fermentation and the dendrogram provides visualization of a hierarchical clustering of experimental conditions. The columns correspond to sRNA expression patterns of samples harvested at different time points during the fermentation indicated in Fig. 1b and ordered consecutively according to time of harvest, batch (point 1), fed-batch (points 2–5), transition (points 6–7) and stationary (points 8–9) phases. Only the eighty-four sRNAs exhibiting significant differential expression are included. A scale of z-score relation to color intensity is shown. **b** The magnitude of expression changes for each differentially expressed sRNA between stationary and batch exponential growth phases is shown. Novel and previously detected sRNAs are represented by blue and red symbols, respectively. The names of some of the sRNAs with the largest fold expression changes are indicated

For differential expression analysis, read counting was performed for each sRNA, followed by normalization and statistical analysis. In the high cell density fermentation, cells were harvested in four different phases of growth, including batch exponential (glucose excess), fed-batch exponential (glucose limitation), transition and stationary phases. A heat map including only the eighty-four sRNAs with significant differential expression reveals four different expression profile types according to the harvest point (Fig. 4a). Among these the batch exponential expression profile is especially distinct. In comparison, the fed-batch exponential profiles show some level of similarity but are much less pronounced, likely due to the approximately five-fold slower growth rate in this phase. There is a marked shift in samples 6 and 7 and these are similar to the stationary phase samples 8 and 9. In the hierarchical clustering, samples 6 and 7 cluster separately from samples 8 and 9 and are therefore likely capturing the transition into stationary phase during high cell density fed-batch fermentation. Two major clusters of sRNAs are evident from the heat map with high expression in either exponential or stationary phase (Fig. 4a). The substantial changes in growth conditions during the fermentation are underscored by the fact that 60 % of the differentially expressed sRNAs show changes that are four-fold or higher.

The sRNAs with significant expression changes during the fermentation are mainly a result of differential expression between batch exponential and stationary phases (Table 2), where seventy-six out of 462 sRNAs (16.5 %) show differential expression between these two growth phases. The largest changes are observed for the SraL (30-fold) and novel ES217 (33-fold) RNAs that are upregulated and the novel ES220 (177-fold) and ES003 (133-fold) RNAs that are downregulated (Fig. 4b). Several known sRNAs show differential expression in stationary phase that is consistent with expression patterns documented in the literature. The CyaR, MicF, MicL, RybA (MntS), RybB, SraL, and SroC RNAs exhibit increased expression [12, 14, 29–32], whereas the IS092 (IsrB), MgrR, Spf, SraG (PsrO), SroG and SroA RNAs exhibit decreased expression [5, 6, 12, 15]. Most of the differentially expressed sRNAs are novel and the specific expression patterns observed suggest that they may play important regulatory roles during growth (Fig. 4b). Further investigation of these could provide valuable information on cellular regulation and their effects on metabolism during fed-batch and high cell density fermentation. The nine sRNAs with differential expression in fed-batch versus batch exponential phases are novel except for SroA and SroC and may have potential utility in the regulation of metabolism in the fed-batch phase. All nine are changed at greater magnitude in the corresponding direction in stationary phase and their differential expression could consequently be an effect of overlapping conditions, such as slow growth or high cell density. The downregulation of SroA and upregulation of SroC in stationary relative to exponential growth phases is consistent with previous data from Northern blots [12]. A total of fifty-four differentially expressed sRNAs are observed between transition and batch exponential phases, where forty-six are also differentially expressed in the same direction in stationary versus batch exponential phases. Eight transcripts are specifically changed in transition phase compared to batch exponential phase, including RyhB and RprA that are downregulated and SroD and C0719 that are upregulated. There are no sRNAs with differential expression between stationary and transition phases, possibly due to the limited number of samples and the more highly variable physiology encountered during the high cell density fed batch fermentation when compared to the exponential growth phases.

**Table 2:** Summary of differentially expressed sRNAs during high cell density fermentation

Growth phases compared	Number of differentially expressed sRNAs			Known upregulated sRNAs	Known downregulated sRNAs
	Total	Up	Down		
Fed-batch exponential vs. Batch exponential	9	5	4	SroC	SroA
Transition vs. Batch exponential	54	29	25	CyaR, C0719, RNA0-365, RybA, RybB, SraL, SroC, SroD	IsrB, IS128, RprA, RyhB, Spf, SroA, Tff
Stationary vs. Batch exponential	76	43	33	CyaR, MicF, MicL, RNA0-365, RybA, RybB, SraL, SroC	IsrB, IS128, MgrR, PsrN, PsrO, Spf, SroA, SroG, Tff

### Differential expression of sRNAs during chemical stress

**Fig. 5:**
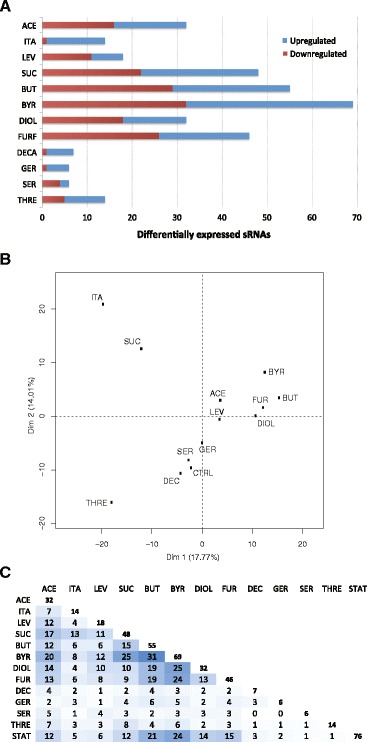
Differential sRNA expression under chemical stress conditions. **a** Bar graph indicating the number of sRNAs whose expression is significantly changed for each chemical stress condition. **b** Principal component analysis. The data shown represent average expression values of three biological replicates for all stress conditions except in the presence of decanoic acid, itaconic acid and threonine, where there is only one replicate. The samples represent chemical stress conditions with the indicated compound, whereas the control (CTRL) is without an added chemical. **c** The number of differentially expressed sRNAs in each stress condition is shown on the diagonal. The other positions show the number of common sRNAs exhibiting differential expression in the same direction under each combination of two stress conditions. Increasing numbers of commonly differentially expressed transcripts are highlighted from light to dark blue

For the 462 identified sRNAs, 138 (29.9 %) are differentially expressed during chemical stress, eighty-four (18.2 %) are differentially expressed during high cell density fermentation, and 177 (38.3 %) are differentially expressed in at least one condition. There are forty-four sRNAs (9.5 %) that exhibit differential expression under both chemical stress and fermentation conditions. The number of differentially expressed sRNAs for each chemical stress condition is shown in Fig. 5a. The numbers of sRNAs with significantly changed expression during chemical stress vary according to the different compound types, where the organic solvents produce the most widespread changes, followed by the acids, and then relatively few changes with the amino acids, decanoic acid and geraniol. Most of the chemicals elicit similar percentages of up- and downregulated sRNAs. However, the differentially expressed sRNAs are mostly upregulated in the case of itaconic acid, decanoic acid and geraniol stress, and downregulated under serine stress. Approximately 33 % of the differentially expressed sRNAs under these conditions show changes that are four-fold or higher.

For the samples exposed to chemical stress, a principal component analysis plot provides an overview of the expression profiles (Fig. 5b). The samples exposed to the organic solvents cluster together, whereas those treated with acids are more spread. A possible reason for the more separate location of the itaconic and succinic acid samples is the high sodium concentration that was introduced as sodium hydroxide in order to neutralize their acidity, thereby adding an osmotic effect. Other samples such as those treated with serine, geraniol and decanoic acid resemble the untreated control sample and also show minor differential expression.

In order to compare the changes in sRNA expression between different conditions, the number of common differentially expressed sRNAs between each pair of stress conditions was scored (Fig. 5c). There is a significant overlap among the differentially expressed sRNAs between the datasets for samples subjected to stress with the organic solvents and on average an organic solvent condition has an overlap of 45 % of its differentially expressed sRNAs with the other organic solvent conditions but only 23 % with the acid conditions. The overlap of differentially expressed sRNAs in the samples treated with acids is smaller in absolute values. However, in relative values (Additional file 4) an acid condition on average shares 43 % and 42 % with other acid and organic solvent conditions, respectively. The relative overlap with stationary phase is for most chemical stress conditions above 33 %. Consequently a great deal of similarity in sRNA expression exists between acid, organic solvent and stationary phase conditions and especially within the group of organic solvent conditions where the overlap is high in both absolute and relative terms. The extent of sRNA differential expression is a possible reflection of the overall cellular effects of the stress and hence the effects of e.g., the organic solvents on the cell could be both extensive and similar.

### Differential sRNA expression in multiple stress conditions

**Fig. 6:**
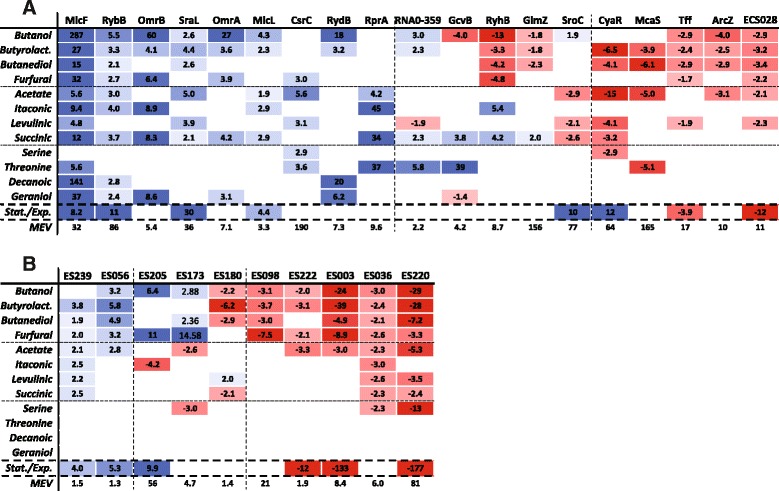
Differential expression of selected sRNAs under chemical stress and fermentation conditions. The selected sRNAs are indicated as columns, whereas the different growth conditions are indicated as rows. The annotated and novel sRNAs with differential expression in at least four chemical stress conditions are shown in panels (**a**) and (**b**), respectively. One novel sRNA (ES205) with differential expression in only three conditions but having a high MEV is also included. The first twelve rows are compound stress conditions relative to a no added compound control, where the compounds are grouped (separated with dashed lines) into organic solvents (row 1–4), organic acids (rows 5–8) and the amino acids, geraniol, and decanoic acid (rows 9–12). The changes in expression during high cell density fermentation in stationary relative to exponential phase are indicated in row 13. Statistically significant differences in expression levels are expressed as fold-changes, with decreases and increases highlighted in red and blue, respectively. The color intensity reflects the magnitude of differential expression. The expression level for each sRNA is represented by the condition-specific MEV (number of reads divided by sRNA length) for the condition with the highest expression for that particular sRNA (row 14). The sRNAs have been grouped according to their differential expression patterns in multiple chemical stress conditions including sRNAs upregulated in multiple conditions (blue only), downregulated in multiple conditions (red only), or mixed expression patterns (blue and red) as described in the text, where the different groups are separated with dashed lines

A group of sRNAs including nineteen known and ten novel transcripts show differential expression in at least four and up to twelve out of thirteen tested stress conditions (Fig. 6). For most of these transcripts, the individual differential expression is unidirectional, especially for conditions within the same chemical group. This is also observed for sRNAs exhibiting changes in both directions, where similar chemicals generally yield expression changes in the same direction. Three overall patterns of differential expression are observed. The first group of transcripts exhibits increased expression in multiple chemical stress conditions (Fig. 6, columns with blue highlighting), where roughly half of these also show increased expression in stationary phase. A second group has decreased expression in chemical stresses, with some also having decreased expression in stationary phase (Fig. 6, columns with red highlighting). A single transcript, CyaR, exhibits decreased expression in chemical stress conditions and increased expression in stationary phase. The third group of transcripts shows unique mixed differential expression patterns under the tested conditions (Fig. 6, columns with blue and red highlighting). In addition to these selected sRNAs, grouping of all differentially expressed sRNAs by hierarchical clustering reveals two overall clusters based on expression in fermentation conditions and six overall clusters based on expression in chemical stress conditions (Additional file 5). The two fermentation-related clusters consist of sRNAs with increased expression in either batch exponential or stationary phases, respectively, while the six chemical stress-related clusters each have their own signature of condition-specific sRNA differential expression.

Several small RNAs of unknown function show differential expression under the tested conditions. Ten hitherto undescribed transcripts show differential expression in four to nine out of thirteen tested stress conditions (Fig. 6b and Table 3). These transcripts are expressed at a level over an MEV of 1 in at least one condition (corresponding to just under 100 reads) and a subset are expressed at higher levels with MEVs between 20 and 82 in at least one growth condition. The expression profiles of these transcripts for the pooled fermentation and stress samples are included in Additional file 6. Most of the sRNA profiles show clearly defined signals compared to the signals of the adjacent genes. For a few transcripts including ES003 the expression profiles from the separate fermentation or stress samples showed a more sharp separation from adjacent genes and these transcripts were therefore assigned as intergenic sRNAs rather than UTRs. All transcripts except two have experimentally determined transcription start sites [27, 33–35] that are consistent with the coordinates defined in this study (Table 3). The ES036 and ES098 transcripts lacking promoter evidence could come from longer processed transcripts and be part of the growing group of sRNAs that are derived from within coding regions, in particular 3′-UTRs [36]. The novel transcripts do not contain predicted Rho-independent terminator sequences. For most of the novel transcripts, sequence conservation is limited to the *Escherichia* and *Shigella* genera, but the ES056 and ES098 transcripts are also conserved in *Citrobacter* and *Salmonella*, respectively (Table 3).

**Table 3:** Selected novel intergenic small RNA transcripts

Name	Start^a^	End^a^	Length^b^	Direction	TSS coordinates^c^	Conservation^d^
ES003	29551	29603	53	+	I	Esc,Shi
ES036	740211	740253	43	+	-	Esc,Shi
ES056	1102475	1102566	92	-	+1	Esc,Shi,Cit
ES098	1814204	1814245	42	+	-	Esc,Shi,Sal
ES173	3154543	3154606	64	+	+8	Esc,Shi
ES180	3295030	3295119	90	-	+11	Esc,Shi
ES205	3657034	3657105	72	+	I	Esc,Shi
ES220	4010909	4011013	105	+	I	Esc,Shi
ES222	4056241	4056348	108	+	−12	Esc,Shi
ES239	4434589	4434711	123	+	I	Esc,Shi

^a^The transcript coordinates determined by RNA seq data

^b^The transcript length determined by RNA seq data

^c^The presence of experimentally determined transcription start sites (TSS) from previous studies is indicated, as well as any differences in TSS coordinates between previous studies and present study. For example ‘+1’ denotes a previously determined TSS one nucleotide downstream of that determined in this study. Identical coordinates are indicated with ‘I’ and cases with no previously determined TSSs or predicted promoters are marked with ‘-’

^d^The sequence conservation of the transcripts in other bacterial genera is indicated: Esc *Escherichia*, Shi *Shigella*, Sal *Salmonella*, Cit *Citrobacter*

### Growth experiments with sRNA mutant strains

Strains overexpressing two of the novel (ES205, ES220) and seven of the previously annotated or predicted (GcvB, McaS, RprA, RydB, RyhB, SraL, SroC) differentially expressed sRNAs, as well as strains with deleted sRNA (for all except GcvB and SraL) were constructed and used in chemical stress growth experiments to measure growth rates compared to wild type controls (Additional file 7). Overexpression was achieved by high copy number plasmid using rhamnose-inducible expression. Three or four chemical concentrations for each compound were employed ranging from the relatively low concentration used for RNA sequencing and up to ten-fold higher concentrations, with the latter often inhibiting growth completely (Additional file 7). However, no significant differences in growth rate were detected in the mutant strains (data not shown). These results indicate that increasing the expression of these sRNAs or deleting them does not result in measurable changes in growth and as a result does not appear to increase the stress tolerance in the conditions tested. It is possible that increased chemical tolerance would be present in a more sensitive assay such as competition experiments or by assaying e.g., survival of chemical stress in stationary phase, a relevant production phase. Redundancy in regulatory mechanisms compensating for engineered changes in expression of the selected sRNA is another possibility.

## Discussion

The expression of many hitherto undescribed small RNA transcripts is reported in this study. In relation to the two other RNA-seq studies in *E. coli*, there is a much greater sequencing depth relative to one study [17] and a comparable sequencing depth to the other study [16]. The former study, with 89395 reads mapping to known sRNAs and 30831 reads mapping to novel sRNAs, employed computational and experimental evidence of transcription initiation combined with RNA-seq data to identify 117 novel intergenic transcripts [17]. The latter study had 3 million reads for annotated sRNAs, 4.3 million reads for novel intergenic regions and reported only 10 novel sRNAs [16]. Although a number of transcripts mapping to intergenic regions with high expression levels were also detected, direct comparison with these is precluded by the fact that only coordinates of entire transcript-containing intergenic regions rather than transcript coordinates are reported [16]. Considering that 117 novel intergenic transcripts were found with low sequencing depth our finding of 253 novel sRNAs with high sequencing depth seems proportionate.

**Fig. 7:**
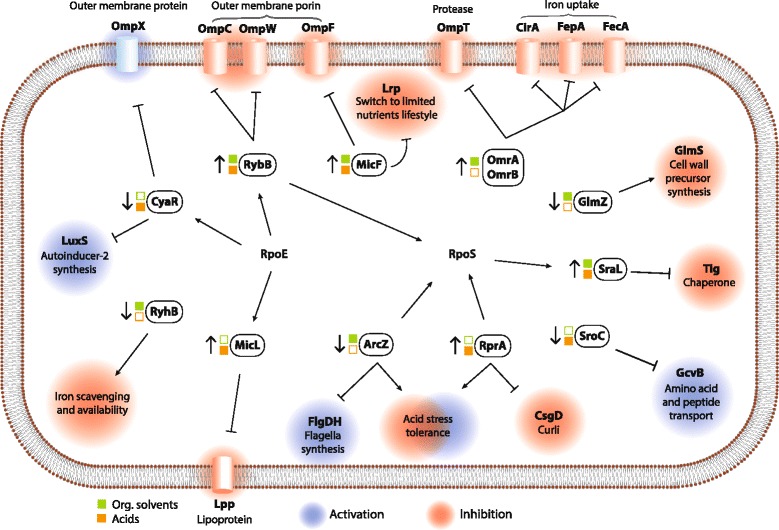
Model of sRNA involvement in the chemical stress response in *E. coli*. An overview of differentially expressed annotated sRNAs during chemical stress with acids and organic solvents. Only sRNAs with significant expression changes in the presence of three or four out of the four acids or four organic solvents are included (filled orange squares, acids; filled green squares, organic solvents). Small RNAs with differential expression in multiple chemical stress conditions are shown with small vertical arrows to indicate increased or decreased expression. The documented action of sRNAs on selected targets or processes is indicated either with blocked lines (repression) or arrows (activation). The potential physiological effects of sRNA differential expression under chemical stress are shown with highlighting, where red indicates inhibition and blue indicates activation. Arrows from the sigma factors RpoE and RpoS indicate the sRNAs with sigma factor dependent expression. The blocked line and arrows from sRNAs to RpoS indicate sRNAs that target the *rpoS* mRNA and result in repression or activation of expression, respectively

There are many examples of sRNAs with expression changes in the same direction under the studied acid and organic solvent stress conditions (Fig. 6). Consequently the differential expression of these sRNAs could mediate similar functional outcomes within but also between the two chemical groups (Fig. 7). For the sRNAs exhibiting differential expression in multiple chemical stress conditions (Fig. 6) three overall groups emerged. These include groups of sRNAs exhibiting solely upregulation, solely downregulation or mixed directions of differential expression in multiple conditions, respectively.

For the group of sRNAs with increased differential expression in multiple stress conditions, several of these are involved in modulating the expression of outer membrane proteins. The MicF and RybB sRNAs are upregulated in nearly all of the 13 tested conditions and modulate the bacterial cell surface by regulating expression of the major outer membrane proteins (OMPs), OmpF and OmpC, which control the transport of molecules into and out of the cell [37]. In particular, RybB is a global regulator that is part of the σ^E^ envelope stress response activation network [38–42] and functions to impart repressor functions to the σ^E^ regulon to maintain envelope homeostasis [43]. As porins comprise only a subset of its targets, the scope of control by RybB extends well beyond the cell envelope and is connected to other global regulatory systems [43]. The MicF sRNA is also known to bind to other targets including the global transcription factor Lrp, where it indirectly activates genes in the Lrp regulon via antisense base-pairing with the translation initiation region of *lrp* mRNA [44]. As Lrp is considered to be an activator of genes required during nutrient-poor conditions and a repressor of genes required in nutrient-rich conditions [44], its potential repression by MicF suggests an increase in energy consumption during chemical stress. The OmrA and OmrB sRNAs are upregulated in five and six chemical stress conditions, respectively, and regulate expression of β-barrel family OMPs including gated channels for iron-siderophore complexes (CirA, FecA, FepA) and the protease OmpT [45]. The newly characterized MicL sRNA is upregulated in five chemical stress conditions and is one of the increasing numbers of sRNAs that originate from within protein-coding rather than intergenic regions only [36, 46]. It is part of the σ^E^ regulon and regulates expression of the abundant outer membrane lipoprotein Lpp by binding to the *lpp* mRNA and inhibiting translation [31]. Taken together, the data indicate that a major physiological outcome mediated by sRNAs under chemical stress is the repression of outer membrane protein expression, likely as a defense mechanism potentially limiting the entry of harmful extracellular molecules.

Other sRNAs with increased expression under multiple chemical stress conditions include the RpoS-dependent SraL RNA, the RpoS-targeting RNA RprA, and the protein-binding CsrC RNA (Figs. 6a and 7). SraL targets the ribosome-associated protein folding chaperone, trigger factor, via binding to the translation initiation region of *tig* mRNA [32]. Upregulation of SraL could function to adjust the level of trigger factor to reduced ribosome and protein synthesis in stationary phase and under chemical stress. RprA activates expression of RpoS by binding to the 5′-UTR of *rpoS* mRNA in an Hfq-dependent manner [47, 48]. The strong increases in RprA expression observed in the presence of acetate, itaconic acid and succinic acid is supported by documented activation of RprA under conditions of cell envelope stress and acidification [49, 50]. Furthermore, expression of RprA has been shown to confer acid resistance when overexpressed together with other RpoS-activating sRNAs, DsrA and ArcZ [49, 51]. The CsrC sRNA binds to and sequesters the mRNA binding protein CsrA via multiple GGA motifs in the loops of predicted stem-loop structures and thereby competes with mRNAs for CsrA binding. Increased CsrC expression in multiple chemical stresses would serve to relieve the effects of CsrA, which represses stationary phase gene expression and activates genes needed for growth [52].

The sRNAs with decreased expression under multiple chemical stress conditions include the CRP-dependent McaS and CyaR RNAs and the RpoS-targeting RNA ArcZ (Figs. 6a and 7). CyaR represses expression of LuxS, NadE, and OmpX by base-pairing with the translation initiation region of target mRNAs [30]. Downregulation of CyaR and possible de-repression of LuxS suggests an increase in quorum sensing during acid stress. McaS is a dual-function RNA that regulates motility and biofilm formation through base-pairing with target mRNAs and via protein binding to the CsrA protein, a negative regulator of e.g., the expression of exopolysaccharide β-1,6 N-acetyl-D-glucosamine (PGA) biosynthesis genes [28, 53]. ArcZ activates RpoS expression by base pairing to the *rpoS* mRNA at overlapping positions targeted by the RprA and DsrA sRNAs [54]. In addition to repression of several specific mRNA targets, overexpression of ArcZ results in differential expression of over 750 genes and loss of motility [55].

Other sRNAs show a mixed response, with increased expression in the presence of some chemicals and decreased expression with others. The RyhB and GlmZ sRNAs are involved in iron and glucosamine-6-phosphate homeostasis [56, 57], respectively, and show similar patterns of mixed differential expression with decreases in the presence of organic solvents and increases in acids (Fig. 7). The combination of upregulation of OmrA/B and downregulation of RyhB suggests a reduction of iron availability during organic solvent stress. The strong increase observed in GcvB expression in the presence of threonine is consistent with its role in regulating expression of amino acid transporters and the *thrL* leader peptide upstream of the threonine biosynthetic operon [58, 59]. A recent investigation has revealed that the SroC RNA functions as an RNA sponge by base pairing with GcvB, triggering its degradation by RNase E, and thereby relieving repression of genes in the GcvB regulon [60]. Their expression is also inversely correlated in the two conditions in which both are differentially expressed.

A number of the sRNAs exhibiting differential expression under chemical stress including OmrA, OmrB, RprA, CsrC, McaS, ArcZ and GcvB are incorporated into complex networks controlling motility and biofilm formation [61, 62]. These regulatory circuits allow sRNAs to integrate extracellular signals to control transcription factor expression and generate appropriate physiological outputs. The mRNAs encoding the RpoS sigma subunit of RNA polymerase and the master regulators of flagella expression (*flhDC*) and curli synthesis (CsgD) have been shown to be targeted by multiple sRNAs and are thus major hubs for sRNA regulation. The above sRNAs regulate the expression of at least one of these three mRNAs via base pairing interactions with the exception of CsrC that acts indirectly via the RNA-binding protein CsrA [54, 63–66].

## Conclusions

In this study, RNA sequencing was used to monitor expression of intergenic small RNAs in the bacterium *E. coli* during high cell density fermentation and chemical stress conditions. A total of 462 transcripts have been investigated, including 253 hitherto unknown transcripts, thereby more than doubling the number of intergenic small RNAs described in this organism. Differential expression analysis of fermentation samples showed that three-quarters of the eighty-four transcripts with significantly changed expression levels are novel, supporting possible regulatory roles for these transcripts in adaptation during different fermentation stages. Further investigation of these could provide new insight into *E. coli* regulation during fed-batch fermentation, which is important for a complete understanding of metabolism that often affects biochemical production. The study has identified twenty-nine novel and previously annotated small RNAs that exhibit differential expression in multiple chemical stress conditions. The effects of differentially expressed sRNAs with known function indicate a general downregulation of many outer membrane proteins, of which many are porins, suggesting a decreased influx of molecules into the cell (Fig. 7). Other effects appear to be decreased availability of iron, RpoS upregulation, as well as an increase in quorum sensing. Overall the differentially expressed sRNAs include several that regulate envelope homeostasis and control bacterial lifestyle, underscoring the involvement of specific sRNAs in coordinating the necessary physiological changes to respond to chemical stress. This work provides insights into sRNAs involved in the chemical stress response and their relevance for engineering in biotechnological applications.

## Methods

### Bacterial strains & growth conditions

The *Escherichia coli* K-12 MG1655 strain was employed in this study. For strain engineering LB medium was used. For chemical stress experiments M9 medium [67] with 0.2 % glucose was used with added trace elements (per liter: 5 mg FeCl_3_ × 6H_2_0, 1 mg ZnSO_4_ × 7H_2_O, 0.2 mg CuCl_2_ × 2H_2_O, 0.5 mg MnSO_4_ × H_2_O, 0.3 mg CoCl_2_ × 6H_2_O, 0.3 mg Na_2_EDTA x 2H_2_O) and vitamins (per liter: 10 μg pyridoxine-HCl, 5 μg thiamine-HCl, 5 μg riboflavin, 5 μg nicotinic acid, 5 μg calcium D-(+)-pantothenate, 5 μg p-aminobenzoic acid, 2 μg biotin, 2 μg lipoic acid, 0.1 μg vitamin B12). For high-cell density fermentation a custom medium was employed containing (per liter): 10 g glucose, 14 g KH_2_PO_4_, 5 g (NH_4_)_2_SO_4_, 2 g citric acid x H_2_O, 1 g MgSO_4_ × 7 H_2_0, 100 μL antifoam (Antifoam A, Sigma), trace elements (per liter: 0.2 g citric acid x H_2_O, 25 mg MnCl_2_ × 4H_2_O, 50 mg NaCl, 75 mg FeCl_3_ × 6H_2_O, 5 mg CoCl_2_ × 6 H_2_O, 25 mg ZnSO_4_ × 7H_2_O, 0.5 mg CuCl_2_ × 2H_2_O, 0.5 mg boric acid, 0.5 mg NaMoO_4_ × 2H_2_O, 5 mg CaCl_2_), vitamins (per liter: 30 mg pyridoxine-HCl, 15 mg thiamine-HCl, 15 mg riboflavin, 15 mg nicotinic acid, 15 mg calcium D-(+)-pantothenate, 15 mg p-aminobenzoic acid, 15 mg lipoic acid, 15 mg biotin, 15 mg vitamin B12). The feed medium for the fed-batch fermentation consisted of 70 % glucose, 10 g/L MgSO_4_, trace elements (per liter: 250 mg citric acid x 2H_2_O, 31 mg MnCl_2_ × 4H_2_O, 62.5 mg, NaCl, 94 mg FeCl_3_ × 6 H_2_O, 62.5 mg CoCl_2_ × 6 H_2_O, 31 mg ZnSO_4_ × 7H_2_O, 0.6 mg CuCl_2_ × 2H_2_O, 0.6 mg boric acid, 0.6 mg NaMoO_4_ × 2H_2_O, 62.5 mg CaCl_2_), vitamins (per liter: 25 mg pyridoxine-HCl, 12.5 mg thiamine-HCl, 12.5 mg riboflavin, 12.5 mg nicotinic acid, 12.5 mg calcium D-(+)-pantothenate, 12.5 mg p-aminobenzoic acid, 12.5 mg lipoic acid, 12.5 mg biotin, 12.5 mg vitamin B12).

For chemical stress experiments cells were grown overnight in M9 medium and diluted to an OD_600_ of 0.05 in 25 mL M9 medium in 250 mL baffled shake flasks. After growth to around OD_600_ 0.9, 25 mL M9 medium with chemical stressor was added and cells were grown for 1 h before harvest. Control cells without chemical stressor in the added 25 mL of M9 medium were grown for approximately 0.5 h to reach the same OD_600_, 0.6-0.7, as the chemically stressed conditions. The employed chemicals were sodium acetate (Sigma, S8750), butanol (Sigma, 281549), 3-hydroxy-butyrolactone (TCI Chemicals, H0939), 1,4-butanediol (Merck, 801534), decanoic acid (Sigma, W236403), furfural (Sigma, 185914), geraniol (TCI Chemicals G0027), itaconic acid (Sigma, I29204), levulinic acid (Sigma, L2009), L-serine (Sigma, S4311), succinic acid (Sigma, S9512) and L-threonine (Sigma, T8441). Triplicates were performed for most compounds except three (decanoic acid, itaconic acid and threonine) to reduce the total number of samples added to the Illumina flow-cell lane. Compounds were selected based on importance and similarity to other compounds.

For high cell density fermentation, cells grown overnight were diluted to an OD of 0.1 in 0.5 L fermentation medium within a 1 L fermentor. At an OD_600_ of approximately 11, glucose was consumed and feed was turned on. During fed-batch fermentation feed was added to sustain an average growth rate of 0.14 h^−1^ corresponding to a doubling time of 5 h. The feed was increased at a steady state while avoiding excess glucose in the medium, thereby keeping cells glucose limited. The pH was monitored and maintained at 6.8 and oxygen saturation was measured and oxygen sparged into the medium during later stages of fed-batch growth to maintain saturation above 50 %.

### Isolation and processing of RNA

RNA isolation and processing was performed as previously described with minor modifications [26]. Briefly, cells were harvested by adding 10 mL of cell culture to 2 mL ethanol containing 5 % phenol, followed by pelleting of cells by centrifugation and freezing at −80 °C. Subsequently cell pellets were lysed with 1 mg/mL lysozyme for 5 min and RNA extracted using Trizol and chloroform. For DNA removal each sample was treated with 40 units DNase I for 30 min. The purity and quality of RNA was verified with spectrophotometer and Bioanalyzer (Agilent Technologies). RNA molecules of length 50 – 500 nucleotides, containing sRNAs, were selected on polyacrylamide-urea gels (Bio-Rad), followed by depletion of 5S rRNA employing the MICROBExpress kit (Life Technologies) using an HPLC purified custom Capture Oligo specific for 5S rRNA (5′-AAAAAAAAAAAAAAAAAAGCGTTTCACTTCTGAGTTCGGCA-3′). RNA was then treated with Tobacco Acid Pyrophosphatase (Epibio) (10 U per sample, containing up to 10 μg RNA) and fragmented using RNase III (Fermentas) for 10 min. Purity and size distribution of RNA was assessed with spectrophotometer and Bioanalyzer after this and the preceding steps. Finally sRNA libraries were constructed using the TruSeq Small RNA Sample Preparation Kit (Illumina) with a few modifications. The protocol was initiated at the section of *Ligate 3′ and 5′ adapters* using 100–400 ng of prepared RNA and was followed up to and including the section *Reverse Transcribe and Amplify*. Subsequently, resulting libraries were purified using AMPure XP beads (Beckmann Coulter). The concentration of the resulting DNA libraries was measured using a Qubit Fluorometer and size distribution assessed using a Bioanalyzer. The libraries were then subjected to paired-end sequencing on an Illumina Hi-Seq 2000 at Beckmann Coulter Genomics.

### Sequencing data analysis

The obtained reads were trimmed using Trimmomatic (standard settings) for removal of low-quality regions of reads. The reads were then mapped to the *E. coli* K-12 MG1655 genome (NC_000913.2) using Bowtie 2 [68] and the resulting sam files were converted to bam files using Samtools [69]. For sRNA identification, bam files for stress samples were merged together, while bam files for fermentation samples were merged together. Using a custom script [26] the number of mapped reads for each nucleotide position in the genome was obtained and intergenic regions containing above 100 reads per nucleotide were selected as potential transcripts. These regions were subsequently manually curated to e.g., join adjacent regions with gaps of few base pairs with fewer than 100 reads per nucleotide or filter out short regions. Potential transcripts were further manually curated by visual inspection using IGV Viewer [70] to remove likely UTRs. In cases where similar expression levels and/or the lack of a gap between the transcript and adjacent gene were observed, the transcript was classified as a UTR and excluded. For differential expression analysis the list of newly identified small RNAs were joined with already annotated sRNAs and previously predicted sRNAs [17]. Read counts for each sRNA in each sample were obtained using CLC Genomics software. Subsequently TMM normalization [71], a scaling normalization method employing weighted trimmed mean of the log expression ratios, was utilized. Differential expression analysis was performed with the edgeR R statistics package [72], regarding counts as a negative binomial distribution and data fitted to generalized linear models. Default parameters were employed and genes with FDR-values below 0.05 were defined as significantly differentially expressed. The mean expression value (MEV) is defined as the number of reads divided by sRNA length. Cumulative MEVs include all reads from all samples, while condition-specific MEVs include only reads from a certain experimental condition. Genome coordinate plot was performed with cgview [73], heatmap and hierarchical clustering employing the Pearson correlation as distance measure with the R packages bioDist and gplots using normalized log-transformed expression values. Principal component analysis was performed with the R package FactoMineR.

### Secondary information on selected novel sRNAs

Promoters of novel sRNAs were either identified from experimental sources [27, 33–35] or predicted using BProm that predicts σ^70^ promoters [74]. Transcriptional terminators were predicted using ARNold [75, 76]. Conservation was estimated by blast analysis towards organisms of the following genera: *Citrobacter*, *Cronobacter*, *Escherichia*, *Edwardsiella*, *Enterobacter*, *Erwinia*, *Klebsiella*, *Pantoea*, *Salmonella*, *Serratia*, *Shigella*, *Sodalis*, *Vibrio* and *Yersinia*. If the identity over the entire length of the sRNA was on average above 90 %, then it was classified as conserved in the respective organism.

### sRNA mutant strain engineering

Briefly, sRNA deletion mutants were constructed using the λ red recombineering technique [77] while sRNA overexpression was performed by insertion of sRNAs downstream of a rhamnose inducible promoter on a pRSFDuet-1 high-copy plasmid. The selected sRNAs were ES205, ES220, GcvB, McaS, RprA, RydB, RyhB, SraL and SroC. For further details see Additional file 7.

### Growth rate inhibition experiments

Growth rates of sRNA deletion mutants were tested using three or four chemical concentrations for each chemical (see Additional file 7) and three biological replicates. Overexpression strains were tested in three or four chemical concentrations using three different rhamnose inducer concentrations in each, but without replicates. Cells were grown overnight in M9 with 0.2 % glucose and diluted into M9 medium with 0.2 % glucose, trace elements, vitamins (the same concentrations as for chemical stress experiments) and relevant chemical added. For overexpression rhamnose was present at transfer. Growth was performed in microtiter 96 square well plates (Enzyscreen B.V.) and these were incubated at 37 °C with 225 rpm shaking in a Growth Profiler 1152 (Enzyscreen B.V.). Growth rates of mutants compared to wild type were tested for statistically significant differences. For sRNA overexpression the control was WT with pRSF-Duet-1 without sRNA insertion.

## Additional files

Additional file 1: **RNA sequencing information.** (XLSX 11 kb)

Additional file 2: **Novel intergenic small RNAs detected in this study.** (XLSX 130 kb)

Additional file 3: **Previously identified intergenic small RNAs.** (XLSX 107 kb)

Additional file 4: **Relative overlap of differentially expressed sRNAs between conditions.** (EPS 1956 kb)

Additional file 5: **Clustering of differentially expressed sRNAs.** (EPS 7438 kb)

Additional file 6: **Expression profiles of selected novel sRNAs.** (EPS 2014 kb)

Additional file 7: **Supplementary Materials and Methods.** (DOCX 31 kb)

## Abbreviations

ace: acetate
but: butanol
byr: 3-hydroxybutyrolactone
deca: decanoic acid
diol: 1,4-butanediol
furf: furfural
ger: geraniol
ita: itaconic acid
lev: levulinic acid
ser: serine
suc: succinic acid
thre: threonine
