# Supplementary material for: Differential expression of small RNAs under chemical stress and fed-batch fermentation in E. coli
Source: BMC Genomics. 2015 Dec 10;16:1051. doi: 10.1186/s12864-015-2231-8 (PMC4676190; doi:10.1186/s12864-015-2231-8)
Supplement: Supplementary file 7 — Supplementary Materials and Methods. (DOCX 31 kb) [file 12864_2015_2231_MOESM7_ESM.docx]

**Additional File 7: Supplementary Materials and Methods**

**Bacterial strains and generation of sRNA mutants**

The employed bacterial strains are presented in Table 1.

Table 1: Bacterial strains

| **Name** | **Strain** |
| --- | --- |
| Wildtype | *E. coli* K-12 MG1655 |
| MHR50 | *E. coli* K-12 MG1655 ΔES205 |
| MHR55 | *E. coli* K-12 MG1655 ΔES220 |
| MHR66 | *E. coli* K-12 MG1655 ΔMcaS |
| MHR68 | *E. coli* K-12 MG1655 ΔRprA |
| MHR54 | *E. coli* K-12 MG1655 ΔRydB |
| MHR70 | *E. coli* K-12 MG1655 ΔRyhB |
| MHR64 | *E. coli* K-12 MG1655 ΔSroC |
| MHR94 | *E. coli* K-12 MG1655 pMHR43 |
| MHR78 | *E. coli* K-12 MG1655 pMHR43 + ES205 |
| MHR79 | *E. coli* K-12 MG1655 pMHR43 + ES220 |
| MHR87 | *E. coli* K-12 MG1655 pMHR43 + McaS |
| MHR89 | *E. coli* K-12 MG1655 pMHR43 + RprA |
| MHR88 | *E. coli* K-12 MG1655 pMHR43 + RydB |
| MHR91 | *E. coli* K-12 MG1655 pMHR43 + RyhB |
| MHR86 | *E. coli* K-12 MG1655 pMHR43 + SroC |
| MHR90 | *E. coli* K-12 MG1655 pMHR43 + GcvB |
| MHR115 | *E. coli* K-12 MG1655 pMHR43 + SraL |

sRNA deletion mutants were constructed using the λ Red recombineering technique [1]. Primers (Table 2) contain the sRNA flanking region and a complementary part of a chloramphenicol resistance cassette from pKD3. The resulting amplification product was used in subsequent recombineering and led to deletion of the entire length of sRNA while maintaining the flanking region with the chloramphenicol cassette inserted in between.

Table 2: Primers for generation of sRNA deletion mutants.

| **Name** | **Sequence** |
| --- | --- |
| KO_ES205_fw | ATGAATAATTTGCGCTTGAGGAATATACAGTAACCGCCAATTATGGATGTGTGTAGGCTGGAGCTGCTTC |
| KO_ES205_rev | CCAGTAAGGTGGGATACAGGCACAGTGATCGACATGGTGAGGTCAACGACATGGGAATTAGCCATGGTCC |
| KO_ES220_fw | ATGAATAATTTGCGCTTGAGGAATATACAGTAACCGCCAATTATGGATGTGTGTAGGCTGGAGCTGCTTC |
| KO_ES220_rev | TCTAATTATATGTAAATCCTATGGATTTTGAATTTAGGGAAGGCGGCAAGATGGGAATTAGCCATGGTCC |
| KO_McaS_fw | TTATGCATGATTATTCATTCACGATATTAATAATGTAACTTATATTTTCGGTGTAGGCTGGAGCTGCTTC |
| KO_McaS_rev | AGTTAAAACTGCATAAAAAAATAGAGTCTGTCGACATCCGCCAGACTCTAATGGGAATTAGCCATGGTCC |
| KO_RprA_fw | ATGAGACGAATCTGATCGACGCAAAAAGTCCGTATGCCTACTATTAGCTCGTGTAGGCTGGAGCTGCTTC |
| KO_RprA_rev | GGTAGCGAAGCGGAAAAATGTTAAAAAAAAGCCCATCGTGGGAGATGGGCATGGGAATTAGCCATGGTCC |
| KO_RydB_fw | AAATAATACTAATCGCAGTTTGTGTTAAAACGGCGGGTTAGCTTTATGAGGTGTAGGCTGGAGCTGCTTC |
| KO_RydB_rev | TTCAGAAATAAGAAAACCCTTAAGTCTGTGCGACACAGGCTTAAGGGTTTATGGGAATTAGCCATGGTCC |
| KO_RyhB_fw | TTTGCAAAAAGTGTTGGACAAGTGCGAATGAGAATGATTATTATTGTCTCGTGTAGGCTGGAGCTGCTTC |
| KO_Ryhb_rev | TAACGAACACAAGCACTCCCGTGGATAAATTGAGAACGAAAGATCAAAAAATGGGAATTAGCCATGGTCC |
| KO_SroC_fw | GTCAGACGAAATGAAAGCACTGTTCAAAGAACCGAATGACAAGGCACTGAGTGTAGGCTGGAGCTGCTTC |
| KO_SroC_rev | GACATAAATCTACTCCAGAAAAAAGAGGGTAGCAGCGTTAACTGCTACCCATGGGAATTAGCCATGGTCC |

sRNA overexpression strains were generated from plasmid pMHR43, which is based on the backbone of pRSFDuet-1. Plasmid pMHR43 contains a kanamycin resistance cassette, the *rhaRS* genes and rhamnose promoter used for rhamnose induction and a terminator. For sRNA overexpression the sRNA sequence is positioned at the transcription start site downstream of the rhamnose promoter and with the terminator located immediately downstream of the sRNA to ensure transcription termination as the novel sRNAs used here might not include a terminator. Briefly, pMHR43 with an sRNA gene insertion was constructed by inserting the sequence for an sgRNA gene [2] containing a strong terminator into the multicloning site in pRSFDuet-1. Next, three DNA parts consisting of the pRSFDuet-1 backbone with terminator; the *rhaRS* genes and rhamnose promoter; and the sRNA sequence were amplified using primers listed in Table 3 and assembled employing USER cloning [3]. Plasmid pMHR43 without a sRNA was constructed assembling the pRSFDuet-1 backbone with terminator (here amplified with the alternative primer for pRSFDuet-1, pRSFDuet1_empty_fw) and the RhaRS genes with rhamnose promoter employing USER cloning with the alternative primer for pRSFDuet-1 (pRSFDuet1_empty_fw). Final plasmids were transformed into *E. coli* K-12 MG1655.

Table 3: Primers employed for generation of overexpression plasmids

| **Name** | **Sequence** |
| --- | --- |
| pRSFDuet1_fw | ATTTGTUTTGAAAAAGTGGCACCGAGT |
| pRSFDuet1_empty_fw | ACTGGTCGUTTGAAAAAGTGGCACCGAGT |
| pRSFDuet1_rev | ACGAACGUATCTCGACCGATGCCCTTGA |
| RhaRS_fw | ACGTTCGUTTAATCTTTCTGCGAATTGAGATGAC |
| RhaRS_rev | ACGACCAGUCTAAAAAGCGC |
| ES205_fw | ACTGGTCGUGTATCCACCAGTAGAACCCT |
| ES205_rev | AACAAAUAAAAAAAATGTTGCCGTTCTG |
| ES220_fw | ACTGGTCGUGTAAACATCTGGACGGCTAA |
| ES220_rev | AACAAAUTATTCATCCCCGGGAGCTTA |
| McaS_fw | ACTGGTCGUACCGGCGCAGAGGAGACAAT |
| McaS_rev | AACAAAUAAAAAAATAGAGTCTGTCGACATCCGC |
| RprA_fw | ACTGGTCGUACGGTTATAAATCAACATATTGAT |
| RprA_rev | AACAAAUAAAAAAAGCCCATCGTGGGA |
| RydB_fw | ACTGGTCGUATTATTCTTATCGCCCCTTCAAGAG |
| RydB_rev | AACAAAUCTACCCCATCCGGCGCTTAt |
| RyhB_fw | ACTGGTCGUGCGATCAGGAAGACCCTCGC |
| Ryhb_rev | AACAAAUAAAAAAAGCCAGCACCCGGC |
| SroC_fw | ACTGGTCGUATTTCGAACTGTCAGACGAA |
| SroC_rev | AACAAAUAAAAAAGAGGGTAGCAGCGT |
| GcvB_fw | ACTGGTCGUACTTCCTGAGCCGGAACGAA |
| GcvB_rev | AACAAAUAAAAAAAGCACCGCAATTAGGC |
| SraL_fw | ACTGGTCGUATCAACACCAACCGGAACCT |
| SraL_rev | AACAAAUAAAACTAAAGCGCCACAAGG |

**Growth rate inhibition experiment**

Nine sRNAs were selected as candidates for improving chemical stress tolerance, two novel (ES205, ES220) and seven annotated (GcvB, McaS, RprA, RydB, RyhB, SraL, SroC). Strains overexpressing each of the nine sRNAs and strains with deleted sRNAs (all except GcvB and SraL) were examined. Growth rates of sRNA deletion mutants were tested using three or four concentrations for each chemical (Table 4) and three biological replicates. Overexpression strains were tested in three or four chemical concentrations (Table 4) using three different rhamnose inducer concentrations (10 μM, 100 μM and 1000 μM) for each, but without replicates. Cells were grown overnight in M9 medium with 0.2 % glucose and diluted into M9 medium with 0.2 % glucose, trace elements, vitamins (the same concentrations as for chemical stress experiments) and relevant chemical added. For overexpression rhamnose was present at transfer. Growth was performed in microtiter 96 square well plates (Enzyscreen B.V.) and these were incubated at 37°C with 225 rpm shaking in a Growth Profiler 1152 (Enzyscreen B.V.). Growth rates of mutants compared to wild type were tested for statistically significant differences. For sRNA overexpression the control was WT with pRSF-Duet1 without sRNA insertion.

Table 4: The four concentrations of each chemical used for growth rate inhibition experiments.

| **Chemical** | **1** | **2** | **3** | **4** |
| --- | --- | --- | --- | --- |
| Acetate (g/L) | 7.5 | 10 | 15 | 20 |
| Butanol (% v/v) | 0.25 | 0.5 | 1 | 2 |
| Butanediol (% v/v) | 2.5 | 5 | 10 | 15 |
| Butyrolactone (% v/v) | 1 | 1.5 | 2 | 3 |
| Decanoic acid (% v/v) | 0.15 | 0.3 | 0.5 | 1 |
| Geraniol (% v/v) | 0.16 | 0.5 | 1 |  |
| Furfural (% v/v) | 0.1 | 0.2 | 0.5 | 1 |
| Itaconic acid (g/L) | 25 | 30 | 40 | 50 |
| Levulinic acid (% v/v) | 0.75 | 1.5 | 2.5 | 5 |
| Serine (g/L) | 1.5 | 2 | 4 | 8 |
| Succinic acid (g/L) | 30 | 40 | 50 | 60 |
| Threonine (g/L) | 5 | 10 | 15 | 30 |

References:

1. Datsenko KA, Wanner BL: **One-step inactivation of chromosomal genes in *E. coli* K-12 using PCR products**. *Proc Natl Acad Sci U S A* 2000, **97**(12):6640-6645.

2. Qi LS, Larson MH, Gilbert LA, Doudna JA, Weissman JS, Arkin AP, Lim WA: **Repurposing CRISPR as an RNA-guided platform for sequence-specific control of gene expression**. *Cell* 2013, **152**(5):1173-1183.

3. Nour-Eldin HH, Hansen BG, Norholm MH, Jensen JK, Halkier BA: **Advancing uracil-excision based cloning towards an ideal technique for cloning PCR fragments**. *Nucleic Acids Res* 2006, **34**(18):e122.
